# Computational Prediction of Protein Function Based on Weighted Mapping of Domains and GO Terms

**DOI:** 10.1155/2014/641469

**Published:** 2014-04-23

**Authors:** Zhixia Teng, Maozu Guo, Qiguo Dai, Chunyu Wang, Jin Li, Xiaoyan Liu

**Affiliations:** ^1^Department of Computer Science and Engineering, Harbin Institute of Technology, Harbin 150001, China; ^2^Department of Information Management and Information System, Northeast Forestry University, Harbin 150001, China; ^3^Department of Statistical Genetics, Harbin Medical University, Harbin 150001, China

## Abstract

In this paper, we propose a novel method, SeekFun, to predict protein function based on weighted mapping of domains and GO terms. Firstly, a weighted mapping of domains and GO terms is constructed according to GO annotations and domain composition of the proteins. The association strength between domain and GO term is weighted by symmetrical conditional probability. Secondly, the mapping is extended along the true paths of the terms based on GO hierarchy. Finally, the terms associated with resident domains are transferred to host protein and real annotations of the host protein are determined by association strengths. Our careful comparisons demonstrate that SeekFun outperforms the concerned methods on most occasions. SeekFun provides a flexible and effective way for protein function prediction. It benefits from the well-constructed mapping of domains and GO terms, as well as the reasonable strategy for inferring annotations of protein from those of its domains.

## 1. Introduction

More and more sequences of proteins are available due to the advanced sequencing technologies, but the biological roles and functions of the proteins are hardly known. As reported by [1], only less than one percent of proteins have been functionally characterized by experiments. In other words, protein sequencing is faster than annotating protein. To fill this gap, a large number of computational methods have been developed to predict protein functions. These methods exploit biological information including amino acid sequence [2–9], genomic context [10–14], protein interaction networks [15–17], protein structure [18–23], microarray [24], and literate to predict protein functions [25, 26]. However, the newly sequenced proteins are often poor in other biological information except the amino acid sequences. Thus, the development of the sequence-based method is crucial and useful for directing further experimental work.

In the past few years, several sequence-based methods [2–9] have been proposed to infer protein functions. These methods annotated the protein with the representative annotations of its homologues. Intuitively, these methods are also called homology-based methods. Usually, the homology-based methods include two stages: searching homologues through BLAST or PSI-BLAST and selecting representative Gene Ontology (GO) terms from annotations of homologues of the unannotated protein. More specifically, Goblet [2] determined the homologues by a predefined threshold of BLAST* e*-value and annotated the unannotated protein with the GO terms of its homologues. GoFigure [3], OntoBlast [4], and Gotcha [5] weighted the GO terms by the BLAST* e*-values and chose GO terms by their weights. PFP [6, 7] made use of both strongly and weakly similar sequences of the query sequence to increase the coverage of functional annotation. ESG [8] exploited cascading homologues of the unannotated protein iteratively to improve the precision of prediction. ConFunc [9] split the homologues into subgroups according to their annotations and then inferred annotations of the unannotated protein from these subgroups. These methods have a positive impact on protein function prediction. However, the homology-based methods may not work when the unannotated protein has low sequence similarity to other annotated sequences or all of its homologues are not annotated. Furthermore, it is also reported that transferring annotations among homologues may easily produce erroneous results [27].

As is known, domain is the conserved sequence and structure in the evolution of proteins, which plays as the stable and independent functional block of proteins [28]. Besides the detailed sequence, domain also carries some important structural information, that is, active site, which is tightly relevant to biological function [21]. Thus, a domain may be a suitable clue to discover the function of proteins. Statistics on UniProt database (released in May, 2013) show that more than sixty percent of proteins have domains. Moreover, domain databases and tools for efficient domain recognition have been developed including Pfam [29], SCOP [30], RPS-BLAST [31], and HMMER [32]. These databases and tools accelerate the analysis of domains in protein. In general, it seemed that inferring functions from resident domains of the protein is feasible and reasonable.

## 2. Related Works

So far, many efforts have been made for discovering functional signals carried by domains. Schug et al. [33] generated rules for function-domain associations based on the intersection of functions assigned to gene products which contain domains at varying levels of sequence similarity. Hayete and Bienkowska [34] designed an automated predictor based on decision tree to assign functions for domains. Mulder et al. [35] mapped GO terms to the domain if all proteins with the given domain do not exist in the set of proteins without the given GO term. Song et al. [36] transferred functions based on alignment of domain content. In analogy with [35], Forslund and Sonnhammer [37] assigned GO term to domain set if and only if all proteins containing the domain set also are annotated with the given GO term. Rentzsch and Orengo [38] transferred annotations in single profile-based sequence cluster. These methods are easily understood and realized, but they are readily misled into making an error-prone prediction by spurious and missing annotations of proteins. Even a single protein missing a valid GO term is enough to mislead the functional inferring about its domains.

In addition, Zhao et al. [39] utilized the protein-domain features, domain-domain interaction, and domain coexisting features to predict domain function. Their work extended the coverage of domain annotation effectively and provided solid foundation for predicting protein function. However, their work mainly paid attention to domain function rather than how the annotation of domain affects protein function. In our work, we focus on how to predict protein function based on domain annotation.

Recently, the probabilistic models have become increasingly popular for their remarkable performance on uncertainty inference. Forslund and Sonnhammer [37] utilized Naïve Bayesian (NB) model for assigning terms to domain set. Nevertheless the Naïve Bayesian model required that domain sets occurrence independently, which does not come with practice. Thus, Forslund et al. had attempted to reduce the dependencies between domain subsets using an averaged contribution from each domain subset. However, the conditional independence assumption may still not hold. Subsequently, Messih et al. [40] designed two models based on NB: one is DRDO that an averaged contribution from each subset which contains the sequential neighboring domains is used to solve the problem of dependency; the other is DRDO-NB which took recurrence and order of domains into consideration. Although computational complexity of DRDO is lower than that of NB, it may still not satisfy the conditional independence assumption. Moreover, all of these methods pruned GO terms of resident domains before they assigned GO terms to the host protein. Thus, some weak functional signals which may be amplified by dependencies between domains are likely to be neglected.

Fang and Gough [41] generalized a dcGO predictor for inferring GO terms associated with individual domains and supradomains based on protein-level GO annotation (GOA) and families of protein. dcGO exploited *P* value to evaluate the association strength (mentioned as relevance in the following sections to simplify) between domain and GO term. Since *P* value only represents the probability of error involved in null hypothesis, it may not be reasonable for estimating the relevance between domain and GO term by *P* value. In other words, *P* value can be used to determine which GO term is related to the given domain from statistical perspective but it is not enough to measure the degree of their relatedness. Thus, an appropriate metric is needed for weighting the relevance between GO term and domain objectively.

In this paper, we design a method to seek functions for proteins (SeekFun) effectively. Under this method, a mapping of GO terms and domains is constructed based on protein-level GOA and domain compositions of proteins. The relevance between domain and GO term is measured by symmetrical conditional probability. Based on the relevance of resident domains and terms, the relevance between host protein and GO terms is computed. Finally, the GO terms with relevance above a predefined threshold are used to annotate the host protein. The performance of SeekFun is validated by a series of experiments. The results suggest that our method is effective and reliable for protein function prediction.

## 3. Methods

### 3.1. Step 1: Construct and Weight Mapping of Domains and GO Terms

It is assumed that the resident domains may be associated with GO terms of the host protein. It is a rough assumption about the relationship between domain and GO term and may result in a large number of false associations. To differentiate the true associations from the false ones, the relevance between domain and GO term need be measured. Judged with this, the true associations will have higher relevance while the false ones will have lower relevance.

As mentioned earlier, *P* value can be used to determine whether the domain is related to the GO term or not. When the *P* value of domain and GO term is larger than the given significance threshold, it is considered that the domain can be annotated with the GO term, and vice versa. However, the larger *P* value does not mean a more tight relationship between domain and GO term. In simple words, *P* value may be not suitable for measuring relevance between domain and GO term. Suppose that *v*_*j*_ represents that the protein containing domain *d*_*j*_ and *u*_*i*_ denotes that the protein plays the function described by GO term go_*i*_. The conditional probability pr(*u*_*i*_ | *v*_*j*_) means the probability of that the protein containing *d*_*j*_ is annotated by go_*i*_. The pr(*u*_*i*_ | *v*_*j*_) can reflect the dependence of go_*i*_ on the *d*_*j*_. Likewise, the pr(*v*_*j*_ | *u*_*i*_) represents the probability of that the protein annotated by go_*i*_ containing the domain *d*_*j*_. The pr(*v*_*j*_ | *u*_*i*_) can reflect the dependence of *d*_*j*_ on the go_*i*_. Thus, it can be inferred that simple conditional probability can reflect relevance between domain and GO term partly but not enough. As (1), symmetrical conditional probability may be appropriate to measure the relevance between GO term go_*i*_ and domain *d*_*j*_, DR(go_*i*_, *d*_*j*_). Consider

(1) $$\begin{array}{c} \text{DR}\left({\text{go}}_{i},d_{j}\right)=\sqrt{\text{pr}\left(u_{i}\;|\;v_{j}\right)\cdot \text{pr}\left(v_{j}u_{i}\right)}. \end{array}$$

Equation (1) means that the relevance between go_*i*_ and *d*_*j*_ is determined jointly by conditional probabilities between *v*_*j*_ and *u*_*i*_. The bigger the probabilities are, the stronger the relevance between them is. Range of the relevance is from 0 to 1. The higher relevance means that the domain is more probably annotated with the term.

Supposed that #prot(go_*i*_) is the number of proteins which are annotated with the go_*i*_, #prot(*d*_*j*_) is the number of proteins which contain *d*_*j*_, and #prot(go_*i*_,  *d*_*j*_) is the number of proteins which have to do with both go_*i*_ and *d*_*j*_. Accordingly, (1) can be transformed into (2). Consider

(2) DR(goi,dj)=#prot(goi,dj)#prot(dj)·#prot(goi,dj)#prot(goi)=#prot(goi,dj)2#prot(dj)·#prot(goi).

### 3.2. Step 2: Transfer GO Terms of Resident Domains to the Host Protein

As is known, GO terms are organized as a directed acyclic graph and may be related to each other. Thus, predicting functions of proteins should take the relationship between GO terms into consideration. GO has a rule called “true path rule”, which defines the terms along the pathway from a given term to the root term that must annotate the protein if the protein is annotated with the given term. And a path upward from the given term to the root term in GO hierarchy is regarded as a true path of the term. Considering the true path rule, the mapping of GO terms and domains is extended along true paths of the GO terms in our method. Traditionally, if a domain is associated with a GO term, it is also associated with all ancestral terms of the GO term with equal relevance. However, it is reported that the semantics of GO terms has differences even if they are parent-child relationship. Thus, the relevance between the domain and each ancestor of the GO term may be different and the semantic differences between GO terms should be considered.

In fact, the organization of GO terms can be regarded as a split-flow semantic system (SFSS). In SFSS, the root term is the source of semantics which can describe the general functions while others represent semantic branches of the root term and illustrate specific functions. So the terms along the true path of the given term have different capabilities to describe the functions. Generally, for a given function, the ancestral term is more likely to describe the given function than its descendants because the semantics of its ancestors is more general and has more power to describe function. It can be explained by semantic coverage of GO term, which can be roughly estimated by the number of its descendants [42].

Based on these analyses, we proposed a novel strategy, namely RSC, to measure the relevance between domain and ancestral term based on semantic coverage. That is, given a term go_*i*_ which is related to the domain *d*_*j*_ with relevance DR(go_*i*_, *d*_*j*_), the relevance between the domain *d*_*j*_ and the ancestral term go_*k*_ of term go_*i*_, can be calculated by (3). In (3), *D*(·) represent the descendant set of the given term and Anc(go_*i*_) consists of the ancestors of the term go_*i*_. Naturally, along the true path, the term which is nearer to root has bigger relevance value with the given domain than others and it is more probably to annotate the host protein.

(3) $$\begin{array}{c} \text{DR}\left(\text{g}{\text{o}}_{k},d_{j}\right)=\frac{\left|D\left(\text{g}{\text{o}}_{k}\right)\right|}{\left|D\left(\text{g}{\text{o}}_{i}\right)\right|}\cdot \text{DR}\left(\text{g}{\text{o}}_{i},d_{j}\right),\;\text{g}{\text{o}}_{k}\in \text{Anc}\left(\text{g}{\text{o}}_{i}\right). \end{array}$$

It is supposed that protein is associated with all GO terms which are related to the resident domains of the protein. The relevance between protein and GO term can be derived from the relevance of the term and resident domains of the protein. For example, if a protein *P* contains a set of domain *D* = {*d*_1_, *d*_2_,…, *d*_*n*_} and DR(go_*i*_, *d*_*j*_) denote the relevance between go_*i*_ and *d*_*j*_, then the relevance between *P* and go_*i*_, PR(go_*i*_, *P*), can be computed by (4). Consider

(4) $$\begin{array}{c} \text{PR}\left(\text{g}{\text{o}}_{\text{i}},P\right)=\underset{d_{j}\in D,1\leq j\leq n}{\max}\text{DR}\left(\text{g}{\text{o}}_{i},d_{j}\right). \end{array}$$

After the extension, each protein is associated with a group of GO terms with strong or weak relevance. To facilitate comparison, the relevance of proteins and terms need be normalized. Each of GO categories should be analyzed, respectively, as they have different biological meanings. For each protein, the relevance between the protein *P* and the root *r* of subontology (GO: 00003674 for molecular function, GO: 00008150 for biological process, and GO: 00005575 for cellular component), PR(*r*, *P*), is used as baseline because the real annotations of proteins must be split from the root in the GO hierarchy. The normalized relevance of go_*i*_ and *P*, NPR(go_*i*_, *P*), can be measured by (5). The relevance has been standardized to scale from 0 to 1. The higher relevance means that the protein is more probably annotated with the term. Consider

(5) $$\begin{array}{c} \text{NPR}\left(\text{g}{\text{o}}_{i},P\right)=\frac{\text{PR}\left(\text{g}{\text{o}}_{i},P\right)}{\text{PR}\left(r,P\right)}. \end{array}$$

Through the above steps, the relevance of proteins and GO terms has been measured already. To select real annotations from candidate annotations, a threshold *t* of relevance need be defined. If the relevance between protein and term is above the predefined threshold* t* and the term is assigned to the protein, and vice versa. In our study, the threshold* t* is about 0.6~0.7 as the proposed model performs well on the given datasets.

## 4. Results and Discussion

### 4.1. Experimental Datasets

Three up-to-date protein subsets of UniProt, Uniref50, SwissProt, and TrEMBL, are selected to evaluate SeekFun. The proteins which are only annotated with GO term inferred from electronic annotations are excluded from the experimental datasets. The SwissPfam database is used to determine the detailed domain composition of proteins. All the datasets are downloaded on May 20, 2013. The details of the experimental datasets are listed in Table 1.

### 4.2. Evaluation Metrics

Consistent with Critical Assessment of Functional Annotations (CAFA) experiments [42], the precision, recall, and* f*-measure are utilized to judge the performance of methods in our experiments. Given a target protein *x* and *K*(*x*) which is a set of known (true) annotations of *x*, the precision of the method at threshold *t* ∈ [0,1], pr(*t*), can be calculated as

(6) $$\begin{array}{c} \text{pr}\left(t\right)=\frac{1}{m\left(t\right)}\underset{x\in S}{\sum }\frac{\left|K\left(x\right)\cap P_{t}\left(x\right)\right|}{\left|P_{t}\left(x\right)\right|}. \end{array}$$

In (6), *P*_*t*_(*x*) is the set of predictive annotations whose relevance with *x* is above* t*.* S* is the target set for testing. *m*(*t*) is the number of proteins which at least has one predictive GO term under given *t*. Similarly, the recall of method at threshold *t*, rc(*t*), can be computed by

(7) $$\begin{array}{c} \text{rc}\left(t\right)=\frac{1}{\left|\text{S}\right|}\underset{x\in S}{\sum }\frac{\left|K\left(x\right)\cap P_{t}\left(x\right)\right|}{\left|K\left(x\right)\right|}. \end{array}$$

The* f*-measure (the harmonic mean of precision and recall) gives an intuitive number for comparisons of the concerned methods. For each method, the maximal value of* f*-measure on the overall threshold of relevance, *F*_max⁡_, is calculated as

(8) $$\begin{array}{c} F_{\max}=\underset{t}{\max}\left\{\frac{2\cdot \text{pr}\left(t\right)\cdot \text{rc}\left(t\right)}{\text{pr}\left(t\right)+\text{rc}\left(t\right)}\right\}. \end{array}$$

Considering the relationships between GO terms, the comparisons are guided by the true path rule. That is, the *K*(*x*) and *P*(*x*) are extended by adding all ancestors of their members to them before comparing.

### 4.3. Comparisons of Relevance Computed by Different Strategies

To illustrate the rationality of weighting strategies, the relevance weighted by symmetrical conditional probability (*R*_SCP_) is compared with those measured by *P* value (*R*_PV_) and traditional conditional probability (*R*_dSCP_). In fact, it is hard to evaluate the relevance between domain and GO term for lacking of the gold standard. To determine appropriate strategies for weighting relevance, some properties of relevance are analysed. A little random noise may make a difference between observed and real datasets and the relevance should be robust on these similar datasets. To simulate similar datasets, a series of subsets of Uniref50, SwissProt, and TrEMBL is constructed by taking nine of their ten equal-size partitions randomly at a time. The calculations of relevance by different strategies are performed on these subdatasets. The varied distributions of relevance on the different datasets may be good evidence for which strategy is more proper for weighting relevance.

**Figure 1:**
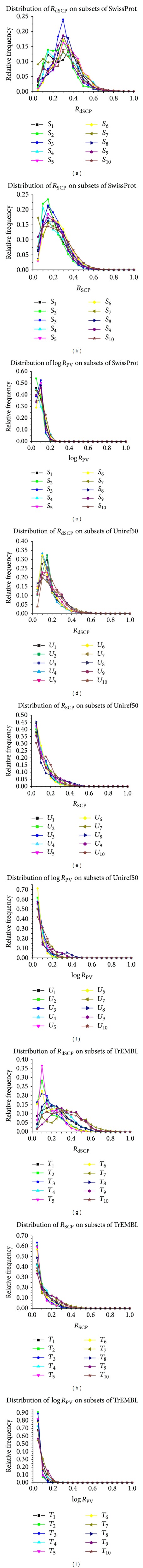
Compare distributions of relevance on similar datasets. *R*_dSCP_, *R*_SCP_, and log⁡*R*_PV_ represent the relevance computed by conditional probability, symmetrical conditional probability, and *P* value, respectively. *S*_*i*_ is constructed by taking nine of ten equal-size partitions of SwissProt at a time, *i* = 1,2 ⋯ 10. Likewise, *U*_*j*_ and *T*_*k*_ denote the constructed subsets of Uniref50 and TrEMBL separately, *j*, *k* = 1,2 ⋯ 10. The curves display the distributions of relevance on similar subsets of the experimental datasets.

The distributions of relevance derived from different strategies are displayed in Figure 1. In order to facilitate comparison, without loss of meanings, the logarithmic transformation and* Z*-score transformation are performed on *R*_PV_, which are represented by log⁡*R*_PV_ in Figure 1. Observed the figure, it can be found that *R*_dSCP_ is the most changeful while the distribution curves of both *R*_SCP_ and log⁡*R*_PV_ have similar trends. All of those suggest that, as for robustness on tiny different datasets, the *R*_SCP_ and *R*_PV_ are more proper than *R*_dSCP_. What is more, the curves of *R*_SCP_ and *R*_PV_ appear to have obvious monotonicity that is beneficial for assigning GO terms to the domain.

Meanwhile, the curves of *R*_PV_ are steeper than those of *R*_SCP_ on each dataset, which imply that the resolution of *R*_SCP_ is lower than *R*_PV_. In this paper, the resolution describes how sensitive the relevance is to distinguish true positive association between domain and GO term from other negative ones. The resolution of relevance is inversely proportional to the average density of relevance in their range, which is just indicated by the steepness of the curves in the figures. In simple words, the larger the average density of relevance in their range is, the harder the true association between domain and GO term is determined.

**Figure 2:**
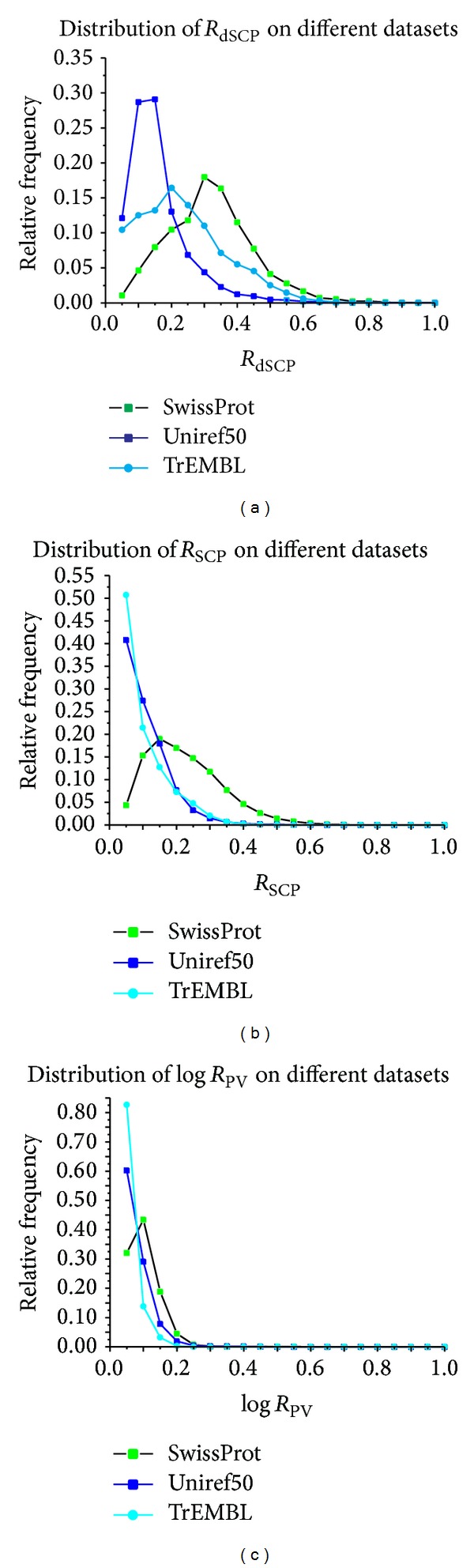
Compare distributions of relevance on significantly different datasets. *R*_dSCP_, *R*_SCP_, and log⁡*R*_PV_ represent the relevance computed by conditional probability, symmetrical conditional probability, and *P* value, respectively. SwissProt, Uniref50, and TrEMBL are the significantly different datasets.The curves display the distributions of relevance on the experimental datasets.

On the other hand, the relevance derived from two significantly different datasets may vary more dramatically than those from the similar datasets. Statistically, the SwissProt and TrEMBL have no intersection while they have 5031 and 6929 common proteins with Uniref50, about up to their 30% and 36% separately. Consequently, the difference between the curves of relevance on SwissProt and TrEMBL should be larger than those of others. Observing the distributions of relevance on these datasets, as displayed by Figure 2, it can be found that the *R*_SCP_ and log⁡*R*_PV_ vary as expected but the log⁡*R*_PV_ still suffers from low resolution. Generally speaking, it can be concluded that *R*_SCP_ is a more suitable measure of relevance between domain and GO term.

### 4.4. The Impact of *R*_SCP_ on Protein Function Prediction

For validating its impact on protein function prediction, *R*_SCP_ is tested on experimental datasets: Uniref50, SwissProt, and TrEMBL, respectively. The comparison is performed on the three subontologies of GO: molecular function (MF), biological process (BP), and cellular component (CC) separately. The comparison includes two steps: constructing mapping of domains and GO terms and annotating proteins based on the mapping.

In our experiment, the mapping of Pfam domains and GO terms (pfam2go) is downloaded from the Gene Ontology website in May, 2013. Based on this reliable mapping, all annotations which are associated with the resident domains are assigned to the host protein. This method is named Pred_pfam2go_ in this paper. Meanwhile, the mapping of Pfam domains and GO terms which is weighted by *R*_SCP_ is also used for prediction, namely, Pred_weighted_. In the comparisons, Pred_pfam2go_ and Pred_weighted_ are validated by performing the same task in the same framework on the basis of different mappings of domains and GO terms. To avoid the influence of domain coverage, the weighted mapping with *R*_SCP_ just includes the domains in pfam2go when it is applied. Here, to compare the influence of the strategy *R*_SCP_ and RSC, the method which is the combination of them is also used to perform the same task and marked with Pred_combine_. Their performances are illustrated in Table 2.

As displayed in Table 2, Pred_weighted_ has higher recall than Pred_pfam2go_ while the latter achieves better precision than the former. These results suggest that the Pred_weighted_ could improve the specificity of annotations but it is at the cost of precision.

It also can be found from Table 2 that Pred_combine_ is superior to others in general. Compared to Pred_pfam2go_, Pred_combine_ outperforms on both precision and recall. In contrast to Pred_weighted_, Pred_combine_ significantly improved the precision while it does as well as Pred_weighted_ on recall. Thus, it can be concluded that *R*_SCP_ tend to select specific terms for the proteins and RSC balances this bias by propagating in the GO hierarchy. It may be the reason that Pred_combine_ shows higher performances.

### 4.5. The Impact of* RSC* on Protein Function Prediction

In order to validate the effectiveness of the* RSC*, it is compared with traditional strategy which set the relevance of domain and terms along a true path as equal (RPE). The two strategies are applied to predict protein functions based on the mapping of domains and GO terms weighted by *R*_SCP_. Their best performances are listed in Table 3.

As displayed, RPE gives a better recall while RSC has higher precision and *F*_max⁡_. In general, RSC may be more beneficial to protein function prediction than RPE. It may be because the resolution of *R*_SCP_ is effectively promoted by different relevance between protein and each term along a true path. On the contrary, RPE considered that protein has equal relatedness to every term along the true path, which makes it harder to determine the true positive associations between terms and the host protein. Even if the threshold of* RPE* is 1, its precision is still lower than the other one and recall goes down. It confirms that the differences of GO terms have significant influence on their relevance with protein.

### 4.6. Comparison of the Concerned Methods

To assess the efficiency of SeekFun, it is compared together with NB, DRDO, DRDO-NB, and dcGO on the three benchmark datasets. The performances of concerned methods on different dataset are shown in Table 4. To provide a simple number for comparison between methods, the averages of metrics on each dataset are also listed.

In terms of precision, SeekFun is superior to others while NB, DRDO, and DRDO-NB follow in turn. The dcGO is significantly lower than others. As aforementioned, dcGO measured relevance between domain and GO term by *P* value while other methods calculated it based on conditional probability. These results may indicate again that the relevance estimated by *P* value is not sensitive enough to determine the true positive associations between domain and GO term. In other words, *R*_PV_ has low resolution for distinguishing real annotations of protein. By contrast, the conditional probability is more suitable for estimating relevance.

As for the recall, SeekFun performs better than others while dcGO follows. It also can be found that the performances of NB, DRDO, and DRDO-NB are not as well as the other methods. Comparing the details of them, NB, DRDO, and DRDO-NB infer functions of protein from annotations of domain combinations, which enhance the precision of function prediction. However, in the process of discovering domain combinations, some slightly weak associations between domain and GO term may be neglected. The resident domains of the host protein may interplay as different combinations to perform different functions. Nevertheless, these methods judge domain combination if the members of the domain combination exist in the protein and the *P* value of their combination is above predefined threshold. It may miss information covered in the potential domain combinations and domain themselves. We guess this may be the reason that these methods show lower recall of functions.

Overall, SeekFun has better performance than others. It can attribute to the weighted mapping of domains and GO terms and the strategy for transferring annotations of resident domains to the host proteins. The weighted mapping can reflect the relationship between domain and GO term properly. The transferring strategy takes both the differences and connections of terms into consideration, which greatly promote its capability of distinguishing real associations of domains and terms from the false ones.

## 5. Conclusions

In this paper, SeekFun is developed for protein function prediction. Instead of using amino acid sequence of protein directly, SeekFun takes the resident domains of proteins and protein-level GOA as clues to annotate proteins. We tested the overall performance of SeekFun and the results suggest that SeekFun is superior to the concerned methods: NB, DRDO, DRDO-NB, and dcGO on precision and recall generally.

Meanwhile the effects of relevance computed by symmetrical conditional probability, (*R*_SCP_) and the strategy for inferring annotations of protein from the annotations of its resident domains (RSC) are validated, respectively. The results of these experiments confirmed that both of them are effective and can promote the performance of protein function prediction. In the proposed method, *R*_SCP_ tend to discover specific functions of protein but it cannot ensure the precision and RSC is used to compensate for the lack of *R*_SCP_. So the combination of them achieves high performances. The main idea of SeekFun could be used to acquire knowledge from other functional ontologies based on different domain resources easily. SeekFun will facilitate the discovery of protein functions and the insights into the biological roles of proteins.

## Figures and Tables

**Table 1:** The details of experimental datasets.

	Uniref50	SwissProt	TrEMBL
Number of annotated proteins	20693	17176	19526
Number of proteins with domains	11673	15810	13588
Number of involved domains	4998	4430	3642
Number of involved GOs	4812	7572	3992

**Table 2:** Compare the impact of *R*_SCP_ on protein function prediction.

		Uniref50			SwissProt			TrEMBL		
		MF	BP	CC	MF	BP	CC	MF	BP	CC
Pred_pfam2go_	Precision	0.5568	0.6094	0.5978	0.4861	0.532	0.5557	0.3856	0.3482	0.3954
	Recall	0.441	0.2888	0.1747	**0.6951**	0.4496	0.2255	0.6176	0.6027	0.2255
	*F*_max⁡_	0.4922	0.3918	0.2704	0.5721	0.4873	0.3208	0.4748	0.4414	0.2872

Pred_weighted_	Precision	0.2979	0.2502	0.1944	0.3514	0.2609	0.2611	0.3472	0.2179	0.2033
	Recall	**0.7805**	**0.6959**	**0.8774**	0.5946	**0.6523**	**0.7603**	**0.7917**	**0.7011**	**0.8213**
	*F*_max⁡_	0.4312	0.3681	0.3183	0.4417	0.3727	0.3887	0.4827	0.3325	0.3259

Pred_combine_	Precision	**0.8506**	**0.8622**	**0.7503**	**0.8543**	**0.8577**	**0.7662**	**0.7641**	**0.7939**	**0.835**
	Recall	0.6971	0.5823	0.7655	0.56	0.4093	0.5984	0.7662	0.6371	0.7309
	*F*_max⁡_	**0.7662**	**0.6951**	**0.7578**	**0.6765**	**0.5542**	**0.672**	**0.7651**	**0.7069**	**0.7795**

The best results are in bold.

**Table 3:** Compare the impact of *RSC* on protein function prediction.

		Uniref50			SwissProt			TrEMBL		
		MF	BP	CC	MF	BP	CC	MF	BP	CC
RPE	Precision	0.2709	0.1582	0.184	0.328	0.2334	0.2866	0.2803	0.1664	0.2131
	Recall	**0.8255**	**0.7195**	**0.901**	**0.6801**	**0.5096**	**0.7807**	**0.8625**	**0.7416**	**0.9**
	*F*_max⁡_	0.4076	0.2575	0.3044	0.4424	0.3195	0.4184	0.4224	0.2709	0.3443

RSC	Precision	**0.8782**	**0.8804**	**0.768**	**0.8529**	**0.8616**	**0.7751**	**0.8064**	**0.8071**	**0.8163**
	Recall	0.7876	0.6856	0.8163	0.5953	0.4294	0.6083	0.8229	0.6985	0.7716
	*F*_max⁡_	**0.8304**	**0.7709**	**0.7914**	**0.7012**	**0.5731**	**0.6816**	**0.8146**	**0.7489**	**0.7933**

The best results are in bold.

**Table 4:** Compare the performances of the concerned methods.

		Uniref50			SwissProt			TrEMBL			Average
		MF	BP	CC	MF	BP	CC	MF	BP	CC	
NB	Precision	0.7778	0.7339	0.7421	0.8362	0.8121	**0.8408**	**0.8977**	**0.8477**	**0.8927**	0.8201
	Recall	0.0428	0.0319	0.0244	0.5012	0.4212	0.3718	0.5086	0.3721	0.4819	0.3062
	*F*_max⁡_	0.0812	0.0612	0.0473	0.6267	0.5547	0.5156	0.6493	0.5172	0.6259	0.4088

DRDO	Precision	0.7716	0.7151	0.7109	0.8232	0.8004	0.8312	0.8644	0.8073	0.8623	0.7985
	Recall	0.1777	0.1385	0.1115	0.5868	0.5023	0.4437	0.5517	0.429	0.5422	0.387
	*F*_max⁡_	0.2888	0.2321	0.1928	0.6852	0.6173	0.5786	0.6735	0.5603	0.6657	0.4994

DRDO-NB	Precision	0.8375	0.6906	0.7439	0.7379	0.7186	0.6766	0.8426	0.8471	0.7512	0.7607
	Recall	0.2094	0.232	0.2695	0.2394	0.2272	0.2633	0.157	0.1502	0.1452	0.2104
	*F*_max⁡_	0.335	0.3474	0.3956	0.3615	0.3452	0.379	0.2647	0.2551	0.2434	0.3252

dcGO	Precision	0.4342	0.3751	0.3014	0.558	0.5253	0.4375	0.3801	0.3473	0.3494	0.412
	Recall	0.6127	0.503	0.6127	**0.605**	**0.4303**	0.5904	0.6692	0.5137	0.6509	0.5764
	*F*_max⁡_	0.5083	0.4297	0.4041	0.5805	0.4731	0.5026	0.4848	0.4144	0.4547	0.4725

SeekFun	Precision	**0.8782**	**0.8804**	**0.7682**	**0.8529**	**0.8616**	0.7751	0.8064	0.8071	0.8163	**0.8274**
	Recall	**0.7876**	**0.6856**	**0.8163**	0.5953	0.4294	**0.6083**	**0.8229**	**0.6985**	**0.7716**	**0.6906**
	*F*_max⁡_	**0.8304**	**0.7709**	**0.7914**	**0.7019**	**0.5731**	**0.6816**	**0.8146**	**0.7489**	**0.7933**	**0.7451**

The best results are in bold.
